# Impact of Maspin Polymorphism rs2289520 G/C and Its Interaction with Gene to Gene, Alcohol Consumption Increase Susceptibility to Oral Cancer Occurrence

**DOI:** 10.1371/journal.pone.0160841

**Published:** 2016-08-15

**Authors:** Po-Yu Yang, Nae-Fang Miao, Chiao-Wen Lin, Ying-Erh Chou, Shun-Fa Yang, Hui-Chuan Huang, Hsiu-Ju Chang, Hsiu-Ting Tsai

**Affiliations:** 1 School of Dentistry, Chung Shan Medical University, Taichung, Taiwan; 2 Department of Dentistry, Chung Shan Medical University Hospital, Taichung, Taiwan; 3 Accelerated Bachelor of Science in Nursing, College of Nursing, Taipei Medical University, Taipei, Taiwan; 4 Institute of Oral Sciences, Chung Shan Medical University, Taichung, Taiwan; 5 School of Medicine, Chung Shan Medical University, Taichung, Taiwan; 6 Department of Medical Research, Chung Shan Medical University Hospital, Taichung, Taiwan; 7 Institute of Medicine, Chung Shan Medical University, Taichung, Taiwan; 8 School of Nursing, College of Nursing, Taipei Medical University, Taipei, Taiwan; 9 Department of Nursing, Taipei Medical University Hospital, Taipei, Taiwan; Duke Cancer Institute, UNITED STATES

## Abstract

**Background:**

The purpose of this study was to identify gene polymorphisms of mammary serine protease inhibitor (Maspin) specific to patients with oral cancer susceptibility and clinicopathological status.

**Methodology/Principal Findings:**

Three single-nucleotide polymorphisms (SNPs) of the Maspin gene from 741 patients with oral cancer and 601 non-cancer controls were analyzed by real-time PCR. The participants with *G/G homozygotes* or with *G/C heterozygotes* of *Maspin rs2289520* polymorphism had a 2.07-fold (p = 0.01) and a 2.01-fold (p = 0.02) risk of developing oral cancer compared to those with *C/C* homozygotes. Moreover, gene-gene interaction increased the risk of oral cancer susceptibility among subjects expose to oral cancer related risk factors, including areca, alcohol, and tobacco consumption.

**Conclusion:**

*G* allele of *Maspin rs2289520* polymorphism may be a factor that increases the susceptibility to oral cancer. The interactions of gene to oral cancer-related environmental risk factors have a synergetic effect that can further enhance oral cancer development.

## Introduction

Oral cancer is lethal and usually causes spacious impairment to the organs involved including lesions of lip, tongue, major salivary glands, gums and adjacent oral cavity tissues, floor of the mouth, tonsils, oropharynx, nasopharynx, hypopharynx and other oral regions, nasal cavity, accessory sinuses, middle ear, and larynx [1]. In Taiwan, the incidence rate of oral cancer is 22.2/100,000, and it is the 6^th^ prevalent malignancy [2] and the 5^th^ leading cause of cancer deaths (8.2/100,000) among Taiwanese [3]. Therefore, more efforts are strongly recommended to look for susceptible individuals for early prevention of oral cancer.

Maspin (mammary serine protease inhibitor), a 42-KDa cytoplasmic protein and commonly known as SERPINB5, has been reported as a tumor suppressor by inhibiting cell proliferation, cell cycle progression, cell motility, invasion, and metastasis [4–9]. Gene expression of Maspin are decreased in gingival cystic keratinizing hyperplasia (CKH) and gingival squamous cell carcinoma (SCC) among rats treated with 10–100 mg/kg 3,3’,4,4’-tetrachloroazobenzene, a carcinogen of dioxin-like compound, compared to controls, and the loss of Maspin expression is correlated with extensive and penetrating lesions [8]. Lower expression of Maspin was also found in cell lines derived from highly invasive human oral squamous cell carcinoma (OSCC) [4, 7]. It was reported that a absent expression of Maspin in tumor cells was significantly positive correlation with lymph node metastasis and invasive progression of OSCC, and patients with high levels of Maspin expression had better survival rates compared to those with low expressions of Maspin [4, 5, 7]. Shpitzer et al. found that the level of Maspin in saliva was significantly decreased among patients with tongue cancer, they suggested detecting salivary Maspin level for diagnosis, prognosis, and post-operative monitoring of oral cancer [10]. We suggested that Maspin plays an important role for modulating the progression of oral cancer.

Genetic polymorphisms are reported to be one of the important risk factors of oral cancer susceptibility [2, 11–13]. The *Maspin* gene is located on chromosome 18q21.3 and encoded by a 7-exon [6]. Three functional gene polymorphisms of *Maspin rs2289519 C/T*, *rs2289520 G/C*, and *rs1455555 A/G* are respectively found in exon-1, exon-5, and exon-7 region [9, 14]. Also, it was found that the polymorphism *Maspin 1022A>G* (*rs1455555 A/G*) results in an amino acid substitution of Val for Leu at amino acid 319 in human *Maspin* gene [14]. We suggested that *Maspin* polymorphisms in exon regions could alter the surface structure or protein levels of Maspin, and considerably affect the individual sensitivity to oral cancer. However, to the best of our knowledge, none of studies investigate the impact of gene polymorphisms of *Maspin rs2289519 C/T*, *rs2289520 G/C*, and *rs1455555 A/G* on the susceptibility of oral cancer. In this study, we recruited 1,342 participants, including 741 patients with oral cancer and 601 healthy people to determine whether genetic variations at these exon regions of *Maspin* and their interaction with oral cancer-related risk factor are associated with the susceptibility to and clinicopathological development of oral cancer among Taiwanese people.

## Materials and Methods

### Subjects and specimen collection

A total of 741 patients who were diagnosed with oral cancer, according to the characteristic criteria of national guidelines for oral cancer between April, 2007 and April, 2015 were recruited as a case group at Chung Shan Medical University Hospital in Taichung and Changhua Christian Hospital in Changhua, Taiwan. Meanwhile, 601 resident area-, race-, and ethnic group-matched healthy individuals were randomly selected from the same geographic area to act as the controls.

The whole blood specimens, collected from healthy controls and oral cancer patients, were placed in tubes containing EDTA and were immediately centrifuged and stored at -80°C. The study was performed with the approval of the Chung Shan Medical University Hospital Institutional Review Board and informed written consent was obtained from each individual.

### Sample Size and Statistical Power

Based on the results of Meng et al.[15], assuming 95% confidence intervals (CIs) and *p* = 0.01 for adjusting potential confounding factors, our sample size has at least 95% power to detect a two-fold increase risk in susceptibility to oral cancer associated with genetic polymorphisms of *Maspin rs 14555555*, *Maspin rs 2289519* and *Maspin rs2289520*.

### Genomic DNA extraction

Genomic DNA was extracted from whole blood samples collected from study subjects by QIAamp DNA blood mini kits (Qiagen, Valencia, USA) according to the manufacture's instructions. DNA was dissolved in TE buffer [10 mM Tris (pH 7.8), 1 mM EDTA] and then quantitated by a measurement of OD_260_. Final preparation was stored at −20°C and used as templates in polymerase chain reaction (PCR).

### Real-time PCR

Allelic discrimination of *rs1455555 A/G*, *rs2289519 C/T*, and *rs2289520 G/C* polymorphisms of the *Maspin* gene was assessed with the ABI StepOne™ Real-Time PCR System (Applied Biosystems, Foster City, CA, USA) and analyzed using SDS vers. 3.0 software (Applied Biosystems), with the TaqMan assay. The final volume for each reaction was 5 μL, containing 2.5 μL TaqMan Genotyping Master Mix, 0.125 μL TaqMan probe mix, and 10 ng genomic DNA. The real-time PCR included an initial denaturation step at 95°C for 10 min, followed by 40 cycles of 95°C for 15 s and 60°C for 1 min.

### Statistical analysis

Hardy–Weinberg equilibrium was assessed using a goodness-of-fit χ^2^ test for biallelic markers and estimated on Excel software. The adjusted odds ratios (AORs) with their 95% confidence intervals (CIs) of the association between genotype frequencies and oral cancer risk as well as clinical characteristics were estimated by multiple logistic regression models after controlling for other covariates. A *P* value <0.05 was considered significant. The data were analyzed on SAS statistical software (Version 9.1, 2005; SAS Institute Inc., Cary, NC).

## Results

In our recruited control group, the frequencies of genetic polymorphisms such as *rs1455555 A/G* (*P* >0.05, χ^2^ value: 0.17), *rs2289519 C/T* (*P* >0.05, χ^2^ value: 0.0004), and *rs2289520 G/C* (*P* >0.05, χ^2^ value: 0.19) were in the Hardy-Weinberg equilibrium.

The study estimated differences of demographical characteristics, such as gender; age; area of residence; race; alcohol, tobacco, and areca consumption between oral cancer patients and controls. A significantly different distribution of gender; age; and alcohol, tobacco, and areca consumption between oral cancer patients and controls was found (Table 1).

**Table 1:** The distributions of demographical characteristics in healthy controls and patients with oral cancer.

Variable	Controls (n = 601) (%)	Patients (n = 741) (%)	p value
**Gender**			
Male	109 (18.1%)	25 (3.4%)	p<0.0001
Female	492 (81.9%)	716 (96.6%)	
**Tobacco consumption**			
No	374 (62.2%)	107 (14.4%)	p<0.0001
Yes	227 (37.8%)	634 (85.6%)	
**Alcohol consumption**			
No	382 (63.6%)	320 (43.2%)	p<0.0001
Yes	219 (36.4%)	421 (56.8%)	
**Areca consumption**			
No	505 (84.0%)	158 (21.3%)	p<0.0001
Yes	96 (16.0%)	583 (78.7%)	
**Age (yrs)**			
≦53	370 (61.6%)	351 (47.4%)	p<0.0001
>53	231 (38.4%)	390 (52.6%)	

An χ^2^ exact tests was used between healthy controls and patients with oral cancer.

**Fig 1:**
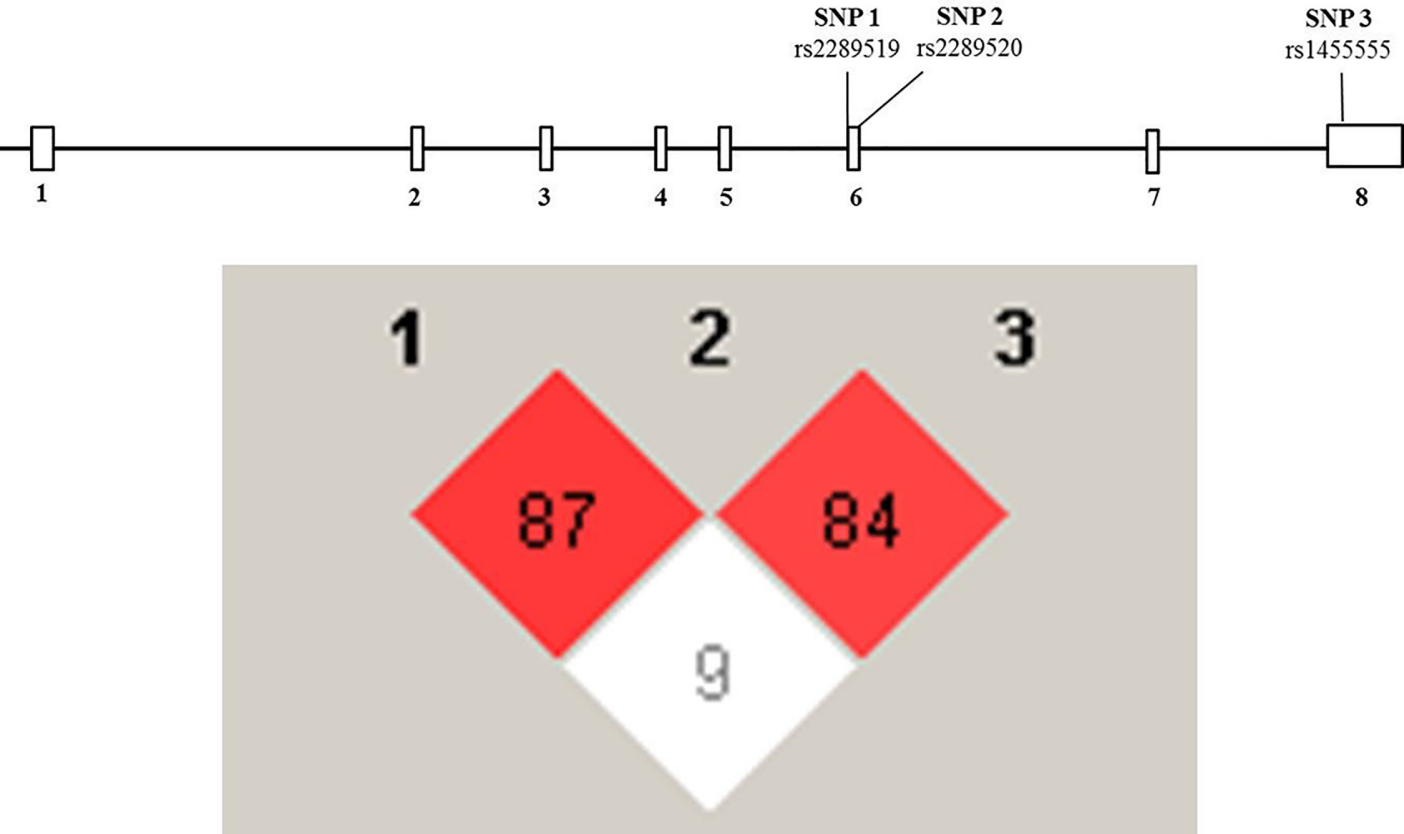
The location of human Maspin gene SNPs their pairwise linkage disequilibrium patterns. Schematic presentation of the Maspin, indicating the locations of the SNP polymorphism. The numbers in the squares represent the pairwise D’ value.

People with *G/G homozygotes* or with *G/C heterozygotes* of *Maspin rs2289520 G/C* polymorphism had a 2.07-fold (95% CI: 1.13–3.77; *P* = 0.01) and a 2.01-fold (95% CI: 1.09–3.70; *P* = 0.02) risk of developing oral cancer compared to those with *C/C homozygotes* after adjusting confound factors. Gene-to-gene interaction effect on the increased susceptibility to oral cancer was also found, the adjusted odd ratios and 95% confidence intervals increased to a 2.46-fold (95% CI = 1.24–4.89; *P* = 0.009) and 2.61-fold (95% CI = 1.30–5.21; *P* = 0.006) risk of developing oral cancer for participants with at least one of the following, including *AG* or *GG* of *rs1455555*, or *CT* or *TT* of *rs2289519*, or *GC* or *GG* of *rs2289520* and for participants with *AG* or *GG* of *rs1455555*, and *CT* or *TT* of *rs2289519*, and *GC* or *GG* of *rs2289520* compared to participants with *AA* of *rs1455555*, and *CC* of *rs2289519*, and *CC* of *rs2289520* (Table 2). The reconstructed linkage disequilibrium plot for the four SNPs was shown in Fig 1. We found that rs2289519 and rs2289520 show a high degree of D’ in our study.

**Table 2:** Adjusted odds ratio (AOR) and 95% confidence intervals (CIs) of oral cancer associated with genotypic frequencies of *Maspin*.

Variable	Controls (n = 601) (%)	Patients (n = 741) (%)	AOR (95% CI)	p value
***Maspin (rs1455555)***				
***AA***	182 (30.3%)	212 (28.6%)	1.00	
***AG***	302 (50.2%)	377 (50.9%)	1.27 (0.90–1.78)	p = 0.16
***GG***	117 (19.5%)	152 (20.5%)	1.14(0.75–1.75)	p = 0.52
***AG or GG***	419 (69.7%)	529 (71.4%)	1.23 (0.89–1.70)	p = 0.20
***Maspin (rs2289519)***				
***CC***	237 (39.4%)	283 (38.2%)	1.00	
***CT***	281 (46.8%)	339 (45.7%)	0.98 (0.71–1.35)	p = 0.93
***TT***	83 (13.8%)	119 (16.1%)	1.18 (0.75–1.84)	p = 0.46
***CT or TT***	364 (60.6%)	458 (61.8%)	1.03 (0.76–1.39)	p = 0.84
***Maspin (rs2289520)***				
***CC***	55 (9.2%)	37 (5.0%)	1.00	
***GC***	246 (40.9%)	284 (38.3%)	2.01 (1.09–3.70)	p = 0.02
***GG***	300 (49.9%)	420 (56.7%)	2.07 (1.13–3.77)	p = 0.01
***GC or GG***	546 (90.8%)	704 (95.0%)	2.18 (1.30–3.65)	p = 0.002
***Maspin* genes combination**				
Group 1	45 (7.5%)	26 (3.5%)	1.00	
Group 2	316 (52.6%)	399 (53.8%)	2.46 (1.24–4.89)	p = 0.009
Group 3	240 (39.9%)	316 (42.7%)	2.61 (1.30–5.21)	p = 0.006

The odds ratios (ORs) with their 95% confidence intervals (CIs) were estimated by logistic regression models. The adjusted odds ratios (AORs) with their 95% confidence intervals (CIs) were estimated by multiple logistic regression models, after controlling for gender, age, alcohol, tobacco, and areca consumption. Group 1: individuals with *AA* of *rs1455555*, and *CC* of *rs2289519*, and *CC* of *rs2289520*; Group 2: individuals with at least one of the following, including *AG* or *GG* of *rs1455555*, or *CT* or *TT* of *rs2289519*, or *GC* or *GG* of *rs2289520*; Group 3: individuals with *AG* or *GG* of *rs1455555*, and *CT* or *TT* of *rs2289519*, and *GC* or *GG* of *rs2289520*.

The study also determined whether there was an interaction effect of gene to related environmental risk-factors on oral cancer susceptibility. The adjusted odd ratios and 95% confidence intervals of genotypic frequencies and oral cancer susceptibility were estimated among persons with exposure and non-exposure to oral cancer-related environmental risk factors, respectively. There was no significant association between genetic polymorphisms of *Maspin rs1455555 A/G*, *rs2289519 C/T*, and *rs2289520* and oral cancer susceptibility among participants who had no exposure to related environmental risk factors (Table 3). However, among alcohol consumers, people with *G/G homozygotes* or with *G/C heterozygotes* of *Maspin rs2289520 G/C* polymorphism had a 4.42-fold (95% CI: 1.80–10.81; *P* = 0.001) and a 3.01-fold (95% CI: 1.22–7.38; *P* = 0.01) increased risk to develop oral cancer compared to those with *C/C* homozygotes. Also, *G/G homozygotes* of *Maspin rs1455555* polymorphism and *T/T homozygotes* of *Maspin rs2289519* polymorphism had a 2.01-fold (95% CI: 1.00–4.05; *P* = 0.04) and a 2.20-fold (95% CI: 1.02–4.73; *P* = 0.04) risk to progress oral cancer among alcohol consumers, after adjusting confounders. Moreover, gene-gene interaction increased the risk of oral cancer susceptibility among subjects expose to oral cancer related risk factors, including areca, alcohol, and tobacco consumption, the adjusted odd ratios and 95% confidence intervals increased to a 3.84-fold (95% CI = 1.41–10.50; *P* = 0.008), 4.48-fold (95% CI = 1.68–11.89; *P* = 0.002), and 2.54-fold (95% CI = 1.06–6.05; *P* = 0.03) risk of developing oral cancer for participants with at least one of the following, including *AG* or *GG* of *rs1455555*, or *CT* or *TT* of *rs2289519*, or *GC* or *GG* of *rs2289520* and a 3.99-fold (95% CI = 1.43–11.10; *P* = 0.007), 6.48-fold (95% CI = 2.35–17.88; *P* = 0.0003), and 2.72-fold (95% CI = 1.13–6.56; *P* = 0.02) for participants with *AG* or *GG* of *rs1455555*, and *CT* or *TT* of *rs2289519*, and *GC* or *GG* of *rs2289520* compared to participants with *AA* of *rs1455555*, and *CC* of *rs2289519*, and *CC* of *rs2289520* when expose to areca, alcohol, and tobacco consumption, respectively (Table 4).

**Table 3:** Adjusted odds ratio (AOR) and 95% confidence intervals (CIs) of oral cancer associated with genotypic frequencies of *Maspin* among individuals non-exposure to related environmental risk factors.

Variable	Controls	Patients	AOR (95% CI)	p value
**Among non-areca consumption (n = 663)**				
***Maspin (rs1455555)***	Control (n = 505) (%)	Case (n = 158) (%)	AOR (95% CI)	p value
***AA***	149 (29.5%)	42 (26.6%)	1.00	
***AG***	258 (51.1%)	86 (54.4%)	1.23 (0.78–1.93)	p = 0.35
***GG***	98 (19.4%)	30 (19.0%)	1.04 (0.59–1.85)	p = 0.87
***AG or GG***	356 (70.5%)	116 (73.4%)	1.18 (0.76–1.81)	p = 0.45
***Maspin (rs2289519)***				
***CC***	202 (40.0%)	60 (37.9%)	1.00	
***CT***	233 (46.1%)	72 (45.6%)	1.09 (0.72–1.66)	p = 0.66
***TT***	70 (13.9%)	26 (16.5%)	1.27 (0.72–2.25)	p = 0.40
***CT or TT***	303 (60.0%)	98 (62.1%)	1.14 (0.76–1.68)	p = 0.51
***Maspin (rs2289520)***				
***CC***	47 (9.3%)	9 (5.7%)	1.00	
***GC***	211 (41.8%)	60 (38.0%)	1.85 (0.81–4.19)	p = 0.13
***GG***	247 (48.9%)	89 (56.3%)	2.07 (0.93–4.62)	p = 0.07
***GC or GG***	458 (90.7%)	149 (94.3%)	1.97 (0.90–4.33)	p = 0.08
***Maspin* genes combination**				
Group 1	37 (7.3%)	7 (4.4%)	1.00	
Group 2	266 (52.7%)	82 (51.9%)	1.97 (0.80–4.87)	p = 0.14
Group 3	202 (40.0%)	69 (43.7%)	2.12 (0.85–5.28)	p = 0.10
**Among non-alcohol consumption (n = 702)**				
***Maspin (rs1455555)***	Control (n = 382) (%)	Case (n = 320) (%)	AOR (95% CI)	p value
***AA***	108 (28.3%)	91 (28.4%)	1.00	
***AG***	191 (50.0%)	170 (53.1%)	1.14 (0.72–1.81)	p = 0.56
***GG***	83 (21.7%)	59 (18.5%)	0.70 (0.39–1.24)	p = 0.22
***AG or GG***	274 (71.7%)	229 (71.6%)	0.99 (0.64–1.53)	p = 0.97
***Maspin (rs2289519)***				
***CC***	139 (36.4%)	116 (36.2%)	1.00	
***CT***	185 (48.4%)	148 (46.3%)	0.77 (0.50–1.19)	p = 0.25
***TT***	58 (15.2%)	56 (17.5%)	0.78 (0.43–1.42)	p = 0.43
***CT or TT***	243 (63.6%)	204 (63.8%)	0.78 (0.51–1.17)	p = 0.23
***Maspin (rs2289520)***				
***CC***	26 (6.8%)	17 (5.3%)	1.00	
***GC***	150 (39.3%)	122 (38.1%)	1.33 (0.57–3.12)	p = 0.50
***GG***	206 (53.9%)	181 (56.6%)	0.95 (0.41–2.18)	p = 0.90
***GC or GG***	356 (93.2%)	303 (94.7%)	1.43 (0.67–3.02)	p = 0.34
***Maspin* genes combination**				
Group 1	22 (5.8%)	12 (3.7%)	1.00	
Group 2	194 (50.8%)	167 (52.2%)	1.41 (0.54–3.68)	p = 0.48
Group 3	166 (43.4%)	141 (44.1%)	1.12 (0.42–2.95)	p = 0.81
**Among non-tobacco consumption (n = 481)**				
***Maspin (rs1455555)***	Control (n = 374) (%)	Case (n = 107) (%)	AOR (95% CI)	p value
***AA***	114 (30.5%)	29 (27.1%)	1.00	
***AG***	190 (50.8%)	60 (56.1%)	1.30 (0.72–2.32)	p = 0.37
***GG***	70 (18.7%)	18 (16.8%)	0.93 (0.43–1.97)	p = 0.84
***AG or GG***	260 (69.5%)	78 (72.9%)	1.19 (0.68–2.07)	p = 0.53
***Maspin (rs2289519)***				
***CC***	143 (38.2%)	42 (39.2%)	1.00	
***CT***	181 (48.4%)	45 (42.1%)	0.83 (0.48–1.43)	p = 0.51
***TT***	50 (13.4%)	20 (18.7%)	1.18 (0.56–2.47)	p = 0.65
***CT or TT***	231 (61.8%)	65 (60.8%)	0.91 (0.54–1.51)	p = 0.72
***Maspin (rs2289520)***				
***GG***	36 (9.6%)	5 (4.7%)	1.00	
***GC***	159 (42.5%)	42 (39.2%)	1.84 (0.60–5.59)	p = 0.27
***CC***	179 (47.9%)	60 (56.1%)	1.90 (0.63–5.72)	p = 0.24
***GC or CC***	338 (90.4%)	102 (95.3%)	2.08 (0.76–5.66)	p = 0.15
***Maspin* genes combination**				
Group 1	29 (7.7%)	4 (3.7%)	1.00	
Group 2	191 (51.1%)	57 (53.3%)	2.03 (0.59–6.95)	p = 0.25
Group 3	154 (41.2%)	46 (43.0%)	1.88 (0.54–6.55)	p = 0.32

The odds ratios (ORs) with their 95% confidence intervals (CIs) were estimated by logistic regression models. The adjusted odds ratios (AORs) with their 95% confidence intervals (CIs) were estimated by multiple logistic regression models, after controlling for gender, age, alcohol, tobacco, and areca consumption. Group 1: individuals with *AA* of *rs1455555*, and *CC* of *rs2289519*, and *CC* of *rs2289520*; Group 2: individuals with at least one of the following, including *AG* or *GG* of *rs1455555*, or *CT* or *TT* of *rs2289519*, or *GC* or *GG* of *rs2289520*; Group 3: individuals with *AG* or *GG* of *rs1455555*, and *CT* or *TT* of *rs2289519*, and *GC* or *GG* of *rs2289520*.

**Table 4:** Adjusted odds ratio (AOR) and 95% confidence intervals (CIs) of oral cancer associated with genotypic frequencies of *Maspin* among individuals exposure to related environmental risk factors.

Variable	Controls	Patients	AOR (95% CI)	p value
**Among areca consumption (n = 679)**				
***Maspin (rs1455555)***	Control (n = 96) (%)	Case (n = 583) (%)	AOR (95% CI)	p value
***AA***	33 (34.4%)	170 (29.2%)	1.00	
***AG***	44 (45.8%)	291 (49.9%)	1.29 (0.76–2.19)	p = 0.32
***GG***	19 (19.8%)	122 (20.9%)	1.29 (0.67–2.48)	p = 0.44
***AG or GG***	63 (65.6%)	413 (70.8%)	1.29 (0.79–2.11)	P = 0.30
***Maspin (rs2289519)***				
***CC***	35 (36.5%)	223 (38.3%)	1.00	
***CT***	48 (50.0%)	267 (45.8%)	0.86 (0.52–1.43)	p = 0.57
***TT***	13 (13.5%)	93 (15.9%)	1.07 (0.52–2.19)	p = 0.85
***CT or TT***	61 (63.5%)	360 (61.7%)	0.91 (0.56–1.46)	p = 0.69
***Maspin (rs2289520)***				
***CC***	8 (8.3%)	28 (4.8%)	1.00	
***GC***	35 (36.5%)	224 (38.4%)	2.38 (0.91–6.17)	p = 0.07
***GG***	53 (55.2%)	331 (56.8%)	2.17 (0.86–5.50)	p = 0.09
***GC or GG***	88 (91.7%)	555 (95.2%)	2.25 (0.91–5.58)	p = 0.07
***Maspin* genes combination**				
Group 1	8 (8.3%)	19 (3.3%)	1.00	
Group 2	50 (52.1%)	317 (54.4%)	3.84 (1.41–10.50)	p = 0.008
Group 3	38 (39.6%)	247 (42.3%)	3.99 (1.43–11.10)	p = 0.007
**Among alcohol consumption (n = 640)**				
***Maspin (rs1455555)***	Control (n = 219) (%)	Case (n = 421) (%)	AOR (95% CI)	p value
***AA***	74 (33.8%)	121 (28.7%)	1.00	
***AG***	111 (50.7%)	207 (49.2%)	1.25 (0.74–2.14)	p = 0.39
***GG***	34 (15.5%)	93 (22.1%)	2.01 (1.00–4.05)	p = 0.04
***AG or GG***	145 (66.2%)	300 (71.3%)	1.42 (0.86–2.36)	p = 0.16
***Maspin (rs2289519)***				
***CC***	98 (44.8%)	167 (39.7%)	1.00	
***CT***	96 (43.8%)	191 (45.4%)	1.28 (0.77–2.11)	p = 0.33
***TT***	25 (11.4%)	63 (14.9%)	2.20 (1.02–4.73)	p = 0.04
***CT or TT***	121 (55.2%)	254 (60.3%)	1.44 (0.90–2.32)	p = 0.12
***Maspin (rs2289520)***				
***CC***	29 (13.3%)	20 (4.7%)	1.00	
***GC***	96 (43.8%)	162 (38.5%)	3.01 (1.22–7.38)	p = 0.01
***GG***	94 (42.9%)	239 (56.8%)	4.42 (1.80–10.81)	p = 0.001
***GC or GG***	190 (86.7%)	401(95.3%)	3.70 (1.56–8.75)	p = 0.002
***Maspin* genes combination**				
Group 1	23 (10.5%)	14 (3.3%)	1.00	
Group 2	122 (55.7%)	232 (55.1%)	4.48 (1.68–11.89)	p = 0.002
Group 3	74 (33.8%)	175 (41.6%)	6.48 (2.35–17.88)	p = 0.0003
**Among tobacco consumption (n = 861)**				
***Maspin (rs1455555)***	Control (n = 227) (%)	Case (n = 634) (%)	OR (95% CI)	AOR (95% CI)
***AA***	68 (30.0%)	183 (28.9%)	1.00	
***AG***	112 (49.3%)	317 (50.0%)	1.10 (0.71–1.71)	p = 0.64
***GG***	47 (20.7%)	134 (21.1%)	1.13 (0.66–1.94)	p = 0.63
***AG or GG***	159 (70.0%)	451 (71.1%)	1.11 (0.74–1.68)	p = 0.59
***Maspin (rs2289519)***				
***CC***	94 (41.4%)	241 (38.0%)	1.00	
***CT***	100 (44.1%)	294 (46.4%)	1.06 (0.71–1.59)	p = 0.76
***TT***	33 (14.5%)	99 (15.6%)	1.19 (0.68–2.11)	p = 0.53
***CT or TT***	133 (58.6%)	393 (62.0%)	1.09 (0.74–1.60)	p = 0.63
***Maspin (rs2289520)***				
***GG***	19 (8.4%)	32 (5.0%)	1.00	
***GC***	87 (38.3%)	242 (38.2%)	2.01 (0.92–4.38)	p = 0.07
***CC***	121 (53.3%)	360 (56.8%)	1.89 (0.88–4.04)	p = 0.09
***GC or CC***	208 (91.6%)	602 (95.0%)	1.94 (0.92–4.07)	p = 0.08
***Maspin* genes combination**				
Group 1	16 (7.0%)	22 (3.5%)	1.00	
Group 2	125 (55.1%)	342 (53.9%)	2.54 (1.06–6.05)	p = 0.03
Group 3	86 (37.9%)	270 (42.6%)	2.72 (1.13–6.56)	p = 0.02

The odds ratios (ORs) with their 95% confidence intervals (CIs) were estimated by logistic regression models. The adjusted odds ratios (AORs) with their 95% confidence intervals (CIs) were estimated by multiple logistic regression models, after controlling for gender, age, alcohol, tobacco, and areca consumption. Group 1: individuals with *AA* of *rs1455555*, and *CC* of *rs2289519*, and *CC* of *rs2289520*; Group 2: individuals with at least one of the following, including *AG* or *GG* of *rs1455555*, or *CT* or *TT* of *rs2289519*, or *GC* or *GG* of *rs2289520*; Group 3: individuals with *AG* or *GG* of *rs1455555*, and *CT* or *TT* of *rs2289519*, and *GC* or *GG* of *rs2289520*.

These genetic polymorphisms were analyzed with regard to the clinical status of each of our recruited 741 oral cancer patients, including the tumor stage, tumor size, lymph node metastasis, distant metastasis, and cancer cell differentiation. There was not a significant association between clinical status and of *Maspin rs1455555 A/G*, *rs2289519 C/T*, and *rs2289520 G/C* gene polymorphism in these patients (data not shown).

## Discussion

To the best of our knowledge, this is the first study to provide novel information of *Maspin rs1455555 A/G*, *rs2289519 C/T*, and *rs2289520 G/C* genetic polymorphism impacts on susceptibility and clinicopathological development of oral cancer.

Genetic factors play pivotal roles in oral cancer susceptibility, and picks oral cancer-related single nucleotide polymorphisms (SNPs) are expected to become the risk markers for early detection of potential candidates for oral cancer [2, 11–13]. Only two studies investigated the role of *Maspin* SNPs in the exon regions for cancer risk [9, 15]. Kim et al. [9] estimated the relationship of *Maspin rs1455555 A/G* and *rs2289520 G/C* genetic polymorphism with susceptibility to gastric cancer. Their study revealed that there was not a significant association between gastric cancer and *Maspin rs1455555 A/G* and *rs2289520 G/C* gene polymorphism [9]. Meng et al.[15] recruited 500 patients with esophageal squamous cell carcinoma and 500 matched controls to estimate the association between SNPs in Serpin gene family and risk of esophageal cancer. They found that *G* allele of *rs2289520 G/C* and *T* allele of *rs2289519 C/T* polymorphisms of *Maspin* were significantly increased the risk of esophageal cancer. In this present study, we found that participants with *G/G homozygotes* (AOR: 2.07; 95% CI: 1.13–3.77; *P* = 0.01) or with *G/C heterozygotes* (AOR: 2.01; 95% CI: 1.09–3.70; *P* = 0.02) of *Maspin rs2289520 G/C* polymorphisms were significantly associated with increased oral cancer risk compared to those with *C/C* homozygotes after adjusting confound factors. In addition, gene-gene interaction to increase oral cancer susceptibility was also found among participants with *AG* or *GG* of *rs1455555 A/G*, *CT* or *TT* of *rs2289519 C/T*, and *GC* or *GG* of *rs2289520 G/C* polymorphisms of *Maspin*. Jang et al. [16] identified a C526T *Maspin* polymorphism in exon 5 from cDNA samples using human cancer cells, which resulted in an amino acid substitution of Ser for Pro at amino acid 176 of Maspin protein. They found that this gene variant induced a significant alteration in the surface structure of Maspin protein and wild-type Pro176 Maspin efficiently induced apoptosis by activating caspase-3 and repressed colony formation of NCI-H157 cells, human lung cancer cell line and decreased tumorigenesis in lung cancer cells in nude mice, but the ability of Ser176 Maspin to stimulate caspase-3 activity was significantly decreased and it was associated with decreased *in vitro* apoptosis and increased *in vivo* tumorigenesis [16]. We suggested that genetic polymorphism of *Maspin rs2289520 G/C* could lead to a lower level or alter structure of Maspin protein [14, 16]. Such an incident or the gene-gene interaction impedes the modulation of cell cycle arrest and the triggering of cell apoptosis, which protects the host from oral cancer development, therefore increases susceptibility to oral cancer [4, 5, 10, 16].

Schwartz et al.[17] found that Streptococci sp and human papilloma virus (HPV) type 16 with exposure to 1% (vol/vol) of ethyl alcohol (ETOH) can play as cofactors in the malignant transformation of oral keratinocytes. Also, it has been reported that ethanol behaves as a solvent in oral mucosa to increase oral cellular membrane penetration to carcinogens and therefore enhances the development of oral cancer [18]. In our study, among alcohol consumers but not for non- alcohol consumers, people with *G/G homozygotes* or with *G/C heterozygotes* of *Maspin rs2289520 G/C* polymorphism had a 4.42-fold (95% CI: 1.80–10.81; *P* = .001) and a 3.01-fold (95% CI: 1.22–7.38; *P* = .01) increased risk to develop oral cancer compared with those with *C/C* homozygotes. Also, *G/G homozygotes* of *Maspin rs1455555* polymorphism and *T/T homozygotes* of *Maspin rs2289519* polymorphism had a 2.01-fold (95% CI: 1.00–4.05; *P* = .04) and a 2.20-fold (95% CI: 1.02–4.73; *P* = .04) increased risk to progress oral after adjusting confounders among alcohol consumers. It was demonstrated that Maspin can reduce cell movement, migration, and invasion by increasing cell adhesion to extracellular matrix molecules [19–21]. We suggested that these genetic polymorphisms in exon region of Maspin, including *G* allele *rs1455555*, *T* allele *rs2289519*, and *G* allele *rs2289520*, could decrease or modulate Maspin protein function, which contributed to a more powerless cell-cell adhesion, and its interaction with alcohol consumption benefited oral cellular penetration to carcinogens and the development of oral cancer.

Moreover, we found that gene to gene interaction increased the risk of oral cancer susceptibility among subjects expose to oral cancer related risk factors, including areca, alcohol, and tobacco consumption, but not among non-exposure. The exposure of people to oral cancer-related environmental risk factors including areca, alcohol, and tobacco consumption show an increased risk to cause mucosal fibroblast proliferation and oral epithelial hyperplasia and dysplasia [18, 22–27]. We suggested that genetic polymorphisms of *Maspin rs1455555 A/G*, *rs2289519 C/T*, and *rs2289520 G/C* could decrease the ability to stimulate apoptosis for mucosal and oral epithelial hyperplasia and dysplasia [16, 18, 22–27]. The inefficiency of induction apoptosis contribute to increase colony formation, moreover, the interaction between gene to gene or gene to related environmental risk-factors help the decrease of tumor suppression and consequently promote the development of oral cancer, particular for subjects expose to areca, alcohol, and tobacco consumption.

One of the limitations of this study is the small sample size. A two stage case-control study design is needed to improve the reliability and reduce the false positive. Therefore, the results should be confirmed by a two stage case-control study with larger population. Furthermore, the functional role of Maspin rs2289520 in cell growth of oral cancer is worth for further investigation, which will be included in our future work. Clones containing various genotypes of Maspin rs2289520 SNPs will be constructed to elucidate the possible functions of Maspin (cell proliferation and cell cycle regulation) in oral cancer cell lines, as well as the underlying mechanisms.

In conclusion, our results suggest that *G* allele of *Maspin rs2289520 G/C* polymorphism may be a factor that increases the susceptibility to oral cancer. The interactions of gene to oral cancer-related environmental risk factors have a synergetic effect that can further enhance oral cancer development.
